# New Horizons of Cementless Total Knee Arthroplasty

**DOI:** 10.3390/jcm13010233

**Published:** 2023-12-30

**Authors:** Giuseppe Polizzotti, Alfredo Lamberti, Fabio Mancino, Andrea Baldini

**Affiliations:** 1 Istituto Chirurgico Ortopedico Traumatologico (ICOT), Sapienza University of Rome, 00185 Rome, Italy; 2 Istituto Fiorentino di Cura e Assistenza, 50139 Florence, Italy; 3 University College London Hospital, London NW1 2BU, UK; 4 The Princess Grace Hospital, London W1U 5NY, UK

**Keywords:** cementless, total knee arthroplasty, survivorship, biomaterials

## Abstract

Background: Considering the increasing number of young and active patients needing TKA, orthopedic surgeons are looking for a long-lasting and physiological bond for the prosthetic implant. Multiple advantages have been associated with cementless fixation including higher preservation of the native bone stock, avoidance of cement debris with subsequent potential third-body wear, and the achievement of a natural bond and osseointegration between the implant and the bone that will provide a durable and stable fixation. Discussion: Innovations in technology and design have helped modern cementless TKA implants to improve dramatically. Better coefficient of friction and reduced Young’s modulus mismatch between the implant and host bone have been related to the use of porous metal surfaces. Moreover, biologically active coatings have been used on modern implants such as periapatite and hydroxyapatite. These factors have increased the potential for ingrowth by reducing micromotion and increasing osteoconductive properties. New materials with better biocompatibility, porosity, and roughness have been introduced to increase implant stability. Conclusions: Innovations in technology and design have helped modern cementless TKA implants improve primary stability in both the femur and tibia. This means that short-term follow-up are comparable to cemented. These positive prognostic factors may lead to a future in which cementless fixation may be considered the gold-standard technique in young and active patients.

## 1. Introduction

Total Knee Arthroplasty (TKA) has been widely recognized as the gold standard treatment for end-stage knee osteoarthritis [1]. This procedure is performed in more than 600,000 patients per year in the United States (US) and the number is projected to remarkably grow by 2030 [2].

The current literature is debatable regarding the efficacy and results of cementless TKA when compared to conventional cemented TKA. It has been often stated that press-fit fixation performs similarly or worse than cemented fixation depending on the selection criteria of patients [3,4]. Moreover, despite several cohort studies detecting comparable outcomes between the two types of fixations, higher costs have limited the widespread diffusion of cementless implants, leaving the conventional technique as the widely recognized gold standard [5,6].

One of the greatest concerns regarding cementless fixation was the increased risk of tibial component early aseptic loosening [7,8,9,10]. However, the development of new implant designs and materials has turned cementless fixation into an interesting and reliable option, especially in younger patients with good bone quality [11]. In addition, radiostereometric analysis (RSA) showed promising results that will be thoroughly analyzed in the following sections [12,13].

Despite the excellent reported outcomes of conventional cemented fixation, young and active patients have been frequently associated with a higher risk for implant revision, refs. [14,15,16] leading to a growing interest in a more durable fixation method.

### 1.1. Mechanical Characteristics of Cementless TKA

Considering the increasing number of young and active patients needing TKA [17], orthopedic surgeons are looking for a long-lasting and physiological bond for the prosthetic implant.

Multiple advantages have been associated with cementless fixation including higher preservation of the native bone stock, avoidance of cement debris with subsequent potential third-body wear, and the achievement of a natural bond and osseointegration between the implant and the bone that will provide a durable and stable fixation. This fixation is based on the migration of osteoblasts and mesenchymal cells towards the implant and the osseointegration through the roughened surface of the implant [18,19]. It has been reported that the minimum requirement for pore size is considered to be approximately 100 µm due to cell size, migration requirements, and transport. However, pore sizes >300 µm are recommended, due to enhanced new bone formation and the formation of capillaries. Moreover, considering that it has been shown that adequate primary stability is a pre-requisite for a successful long-term fixation of uncemented implants [20], a rough surface has a double effect: firstly, on primary stability by increasing the shear-load bearing capacity at the bone-implant interface in the direct post-operative period [21,22], and secondly, on secondary fixation providing a mechanical interlock between bone and implant [22]. However, careful attention must be taken when using a highly rough surface because of potential complications related to the surgical procedure such as required higher insertion forces that may lead to periprosthetic fracture [23] and malseating of the implant [24,25]. Indeed, primary fixation remains crucial in both influencing long-term fixation [26] and in achieving osseointegration by limiting the amount of micromotions [27,28].

### 1.2. First Generation of Cementless TKA

First, the generation of cementless TKA has been associated with numerous design flaws that led to early failure. When evaluating the prosthetic components, the femoral component reached better outcomes than the tibial and patellar counterparts. Femoral component failures were mainly associated with fatigue fractures of the thin areas [29]. Moreover, other pitfalls included the use of sintered beads or mesh coating, non-continuous fixation surfaces, short pegs, poor polyethylene locking mechanisms, and sterilization methods, and the use of metal-backed patellar components that showed poor survivorship [9].

### 1.3. New Materials

Innovations in technology and design have helped modern cementless TKA implants improve dramatically. The better coefficient of friction and reduced Young’s modulus mismatch between the implant and host bone have been related to the use of porous metal surfaces. Moreover, biologically active coatings have been used on modern implants such as periapatite and hydroxyapatite. These factors have increased the potential for ingrowth by reducing micromotion and increasing osteoconductive properties. New materials with better biocompatibility, porosity, and roughness have been introduced to increase implant stability.

#### 1.3.1. Hydroxyapatite

Hydroxyapatite (HA) represented a promising material with the potential to achieve biological fixation of implants. HA coating, in comparison to press fit fixation or porous coating, is an osteoconductive calcium phosphate molecule that can encourage the biological growth of the bone even in the presence of gaps or partially unstable conditions [30]. Moreover, similar micromotions at one to two years have been reported between HA-augmented and cemented implants [31]. In addition, several clinical studies have shown reliable fixation of HA-coated implants in TKA [32,33]. Nelissen et al. [33] compared HA-coated cementless implants with non-coated and cemented TKA reporting better performance in terms of micromotion in the HA and cemented groups in the longitudinal, transverse, and sagittal axes. Similarly, Cross et al. [32], reported on 1000 HA-coated cementless TKA with a cumulative survivorship at ten years of 99% (95% confidence interval [CI] 92.5 to 99.8), supporting the reliability of HA in cementless TKA. Finally, Voigt et al. [34], after evaluating 14 randomized controlled trials (RCT), stated that in patients < 70 years of age, an HA-coated tibial implant may provide better durability than other forms of tibial fixation.

#### 1.3.2. Trabecular Metal

**Figure 1:**
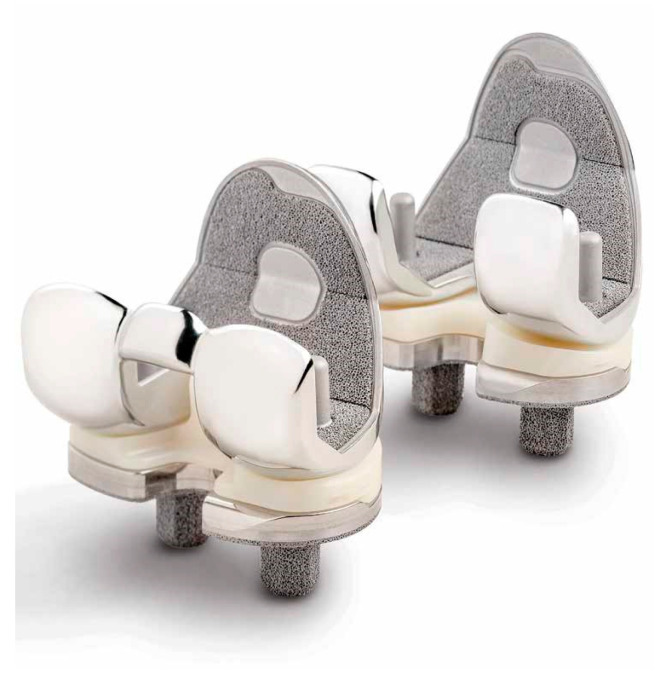
Persona Posterior Stabilized and Medially Congruent cementless with tantalum coating (Zimmer Biomet Inc., Warsaw, IN, USA).

More recently, Trabecular Metal™ (Zimmer Inc., Warsaw, IN, USA) (Figure 1), a newer biomaterial made of tantalum, has been introduced as being similar in porosity to cancellous bone. It has been extensively associated with excellent mechanical and biological properties, including predictable ingrowth and osseointegration, primary stability, and maintenance of bone mineral density (BMD). However, clinical results at the mid-to-long-term follow-up with tibial monoblock components have been controversial [35,36,37,38]. In a recent meta-analysis by Hu et al. [11] on six studies involving 977 patients, the authors stated that the use of cementless porous tantalum monoblock tibial component achieved no substantial superiority over conventional cemented modular tibia at the 5-year follow-up. However, excellent mid-term outcomes have been reported by Niemeläinen et al. [39] on 1143 primary cementless TKAs based on the Finnish Arthroplasty Registry at a mean of 7 years follow-up. The authors reported a survivorship of 100% (95% CI 99–100) at 1, 5, and 7 years postoperatively using revision for aseptic loosening of the tibial component as an endpoint in a population-based setting.

To the best of the author’s knowledge, only a few RCTs have been performed at different follow-ups. Dunbar et al. [40] compared the outcomes of porous monoblock and cemented tibial components in 70 randomized patients at a 24-month follow-up. A subset of the TM components migrated extensively in the postoperative period, but all stabilized by one year with 0.0 implants (95% confidence interval, 0.0 to 0.12) considered to be at risk for early aseptic loosening, whilst four cemented components were considered to be at risk (proportion at risk, 0.19; 95% confidence interval, 0.08 to 0.4). The same cohort of patients was then re-evaluated at a 5-year follow-up [12], reporting similar tibial motions between the 2 groups (*p* = 0.9) and a similar proportion of implants “at risk” (2 of 18 in the cemented group and 0 of 27 in the TM group; *p* = 0.2), suggesting that the TM implants provide solid fixation at mid-term follow-up despite high levels of initial migration. Moreover, Fernandez-Fairen et al. [41], randomized 145 patients into two groups receiving either a TM cementless tibial component or a cemented conventional one reporting similar outcomes at a 5-year follow-up in terms of clinical scores, complication rate, and survivorship from aseptic loosening. Similarly, Pulido et al. [42] randomized 397 patients and evaluated the outcomes at a 5-year follow-up reporting that no highly porous metal tibial components were revised for aseptic loosening and that they provided similar durable fixation and reliable pain relief and restoration of function when compared with a traditional cemented modular tibia in TKA. Finally, Hampton et al. [43] randomized 90 patients into receiving either cementless TKA with TM monoblock tibial component or hybrid fixation TKA at an up-to-15-year follow-up and reported better clinical outcomes (*p* = 0.001) and better radiological analysis compared with the cemented group (*p* < 0.001) despite both groups having excellent survivorship at the final follow-up.

Recently, modular trabecular metal tibial components have become available for clinical use. Fricka et al. [44] randomized 100 patients to receive either the cementless or cemented version (50 patients each) and evaluated survivorship and clinical outcomes at a 2-year follow-up. Despite comparable results, one implant in the cementless group was revised due to implant-related failure; moreover, four other implants experienced a mean 3° varus subsidence and further stabilized with clear signs of osseointegration, while 15% of the cementless implants (7 out of 47) reported some radiolucencies (RLL). The authors assumed that the higher rate of RLL with respect to the non-modular design was probably related to the stiffer titanium baseplate and inflexibility as compared to the flexibility of the metaphyseal bone, suggesting further evaluation to determine their long-term stability.

#### 1.3.3. BIOFOAM

BIOFOAM (Microport Orthopedics, Inc., Arlington, TN, USA) is a cancellous titanium foam that can be manufactured to reach a porosity of up to 80% to increase mechanical properties. Cancellous titanium is a porous reticulated titanium material developed for load-bearing orthopedic implants with a compressive modulus similar to bone and it shows improved material properties with increased porosity and friction coefficient which enhances early stability and osseointegration [45]. Promising short-term outcomes have been reported by Waddell et al. [46] in a retrospective cohort of patients with no cases of implant-related failures and no progressive radiolucencies at 24-month follow-up. Further analysis has been described by Karachalios et al. [45], who retrospectively evaluated two groups of 54 patients treated with cemented and titanium cancellous-foam cementless implants and reported comparable results at a 9-year follow-up, with no cases of implant-related failures in the cementless group and satisfactory radiological outcomes.

#### 1.3.4. Tritanium

A novel modular cementless tibial component (Triathlon^®^ Tritanium^®^, Stryker Orthopedics, Mahwah, NJ, USA) has been introduced. It is made up of a highly porous titanium coating applied by 3-dimensional printing to create a biological fixation surface with a triangular keel and 4 cruciform 9-mm-long pegs coated solely at the base of each peg. This device has been compared in a cadaveric study with a two-peg TM monoblock baseplate reporting reduced rocking motions and liftoff, supporting higher potentials for biological fixation [47]. Clinical results on the same prosthetic implant have been retrospectively reported by Miller et al. [6] on 400 patients with a revision rate due to aseptic loosening of 0.5% at a minimum 2-year follow-up (comparable to the cemented control group) with areas of increased bone density at the pegs of the tibial baseplate. In addition, the same Tritanium implant has been tested in a consecutive series of 406 primary cementless TKA in obese patients and matched 1:1 with a group of the same cemented implant, reporting a high 7-year survivorship free from aseptic revision (99.0% vs. 99.5%, *p* = 0.665) [48]. Nam et al. [29], prospectively randomized 147 patients (67 cemented and 80 cementless) and evaluated the outcomes at a mean 2-year follow-up. The authors reported comparable early outcomes in terms of clinical scores and survivorship with no signs of progressive radiolucencies or component subsidence in either group.

### 1.4. Implant Migration and RSA Analysis

Radiostereometric analysis (RSA) represents a valid method to evaluate implant fixation to bone and early migration, especially within the first two postoperative years, providing a prediction to long-term outcomes. Cementless fixation has shown a pattern of high initial migration called “settling”, followed by stabilization after approximately one year, compared with lower initial migration for cemented components [49]. However, cemented fixation can be affected by late degenerative processes to the cement mantle such as delamination that can compromise implant fixation [5]. Moreover, Pijls et al. [49] reported a clinically relevant association between early migration, as measured with RSA, and long-term clinical failure resulting in revision for aseptic loosening, stating that each mm of migration was associated with an increase in the 5-year revision rate of 8%.

Laende et al. [50] compared the long-term migration of 79 patients with cemented (58 TKA) and cementless (21 TKA) tibial components at a mean of 12 years postoperatively. The authors reported a significant correlation between one-year and long-term migration, especially for cementless components. In addition, the long-term migration was comparable but the inducible displacement (single-leg stance weight bearing) at 10 years was significantly higher for the cemented components (0.2 [range, 0.2–0.4] vs. 0.1 [range, 0.1–0.2]; *p* < 0.001), suggesting at least equivalent, if not superior, long-term fixation of the press-fit technique. Similar findings were detected by Henricson et al. [51] in their RSA analysis at 10 years postoperatively between 26 TM tibial monoblock implants and 21 cemented counterparts. The authors reported that TM implants continued to be firmly fixed to bone at the final follow-up, with stabilization from 3 months onwards after the early initial migration, suggesting that the pattern of migration represents a more reliable factor for analysis of the implant fixation than the magnitude of fixation itself. Therefore, stabilization after the initial settling should be considered as a positive sign for long-lasting fixation, conversely to continuous migration which represents an unfavorable sign. The magnitude of migration at 1 year postoperatively should not be considered as an indicator of potential future loosening of cementless implants. Similarly, Hasan et al. [52] evaluated the RSA analysis of the novel 3D-printed highly porous Tritanium implants, randomizing 72 patients to receive either a cementless (35 patients) or cemented (34 patients) TKA. One 71-year-old female had to be revised for migration of the tibial component 20 months postoperatively in the cementless group, however, despite a higher migration in the first three months, all the press-fit implants resulted well stabilized at a two-year evaluation. Conversely, three cemented implants were initially stable but showed continuous migration between one and two years of follow-up. In addition, the authors reported that the novel 3D-printed cementless TKA showed promising results as the initial migration seemed to be lower than other cementless designs, probably due to the additional four pegs of the baseplate design.

### 1.5. Implant Loosening in Obese and Young Patients

The increasing interest in cementless TKA is additionally related to the higher failure rate of cemented implants in particular subcategories of patients such as young, obese, and active. The mechanisms of failure in obese patients are believed to be related with increased sheer forces and stress at the bone–cement interface, leading to micromotion and aseptic loosening or osteolysis [53]. Whiteside and Viganò [54], reported on a first cementless generation implant comparing the outcomes at a mean of 7 years follow-up of 122 young and heavy patients (<55 years, >90 kg) with 122 older and lighter ones (>65 years, <80 kg), showing no cases of implant loosening and no difference of implant survivorship, suggesting that press-fit fixation is safe in young, overweight patients. In addition, Bagsby et al. [53] retrospectively compared the outcomes in 292 morbidly obese patients (BMI > 40), 154 cemented TKA and 245 cementless. When evaluating aseptic revisions, the authors reported a statistically significant higher incidence of aseptic loosening in the cemented cohort (5.8%) compared with the cementless cohort (9 vs. 0 TKAs, *p* 1/4 0.005) at a mean follow-up of 6.1 years in the former and 3.6 in the latter. Therefore, the authors suggested that cementless fixation may provide biologic bony ingrowth and a subsequent more durable implant–bone interface, which may better tolerate the added mechanical stress generated in this population. Similarly, Sinicrope et al. [55] retrospectively compared 108 cementless TKA with 85 cemented, all in morbidly obese patients (BMI > 40). The authors noted survivorship with aseptic loosening as the endpoint of 99.1% (1 failure) in the former and 88.2% (16 failures) in the latter at a 8-year follow-up (*p* = 0.02), suggesting that cementless fixation may represent a promising alternative to mechanical cement fixation in this category of patients.

Regarding the outcomes in young patients (<55 years), Kim et al. [56] compared, in a prospective high-quality RCT, cemented and cementless implants in bilateral, sequential, and simultaneous TKAs in 80 patients at a mean follow-up of 16.6 years using a first generation cementless device. The authors noted comparable results in terms of clinical outcomes and implant survivorship with one (1.3%) reported case of early mechanical failure (within the first year) in the cementless group. However, the difference was not significant, suggesting a reliable survivorship in young patients in the long-term for both investigated implants. Furthermore, the same group of authors [57] reported a mean follow-up of 23.8 years of 261 patients (522 knees) which randomly underwent simultaneous bilateral TKA with cementless and cemented implants and reported comparable outcomes with 97% and 98% survivorship, respectively. The prevalence of aseptic loosening and osteolysis were similar in both groups, suggesting no substantial differences between the two fixation techniques.

### 1.6. Best Biology for Secondary Fixation

It has been reported that thermal injury to bone is time and temperature dependent, with temperatures below 44° not being associated with osseous injury but with temperatures between 47° and 50° that are maintained for more than 60 s being associated with bone reabsorption and osteonecrosis, increasing the risk of early migration and subsequent failure [49]. A cadaveric study by Vertullo et al. [58] showed that the modern tibial cementing technique has been associated with temperatures below the safety cutoff, despite the narrow thermal safety margin for osseous injury of 4.95° (95% CI ± 4.31) and that cement penetration depth did not correlate with the maximum cement temperature. Moreover, besides the thermal damage potentially generated by cement polymerization, thermal osteonecrosis could be induced by the heat generated by cutting tools such as a saw or burr. Tawy et al. [59] reported in a cadaveric study that mean bone temperatures above 47° were maintained for more than 60 s in non-irrigated bone as well as in bone burred with room temperature irrigation, while uncooled irrigation was effective in reducing the mean temperature of sawed bone to <47° (*p* < 0.05) and the usage of cooled irrigation would prevent the bone from reaching temperatures beyond 47°, either in burred or sawed. Therefore, the authors suggested that irrigation with saline solution at room temperature is effective in reducing the likelihood of thermal osteonecrosis in sawed bone.

### 1.7. Our Surgical Technique Tips

We start with tibia resection, using a 1.27 mm saw blade and we irrigate it with saline water at room temperature, while also trying not to spend so much time on the resection in order to not warm up the bone. In our opinion, reducing the time for cutting, blade thickness, irrigation with saline water, and bone pre-cooling are very good tips to not overheat the bone. Minimizing heat shock is important because thermal necrosis at 60° can cause an immediate cellular depletion and a slow cell recovery [60]. After the resection, the tibia plane should be symmetric and flat. Distal femur resection is performed with the same irrigation and sawing technique. Once again, the flatness of the surface is primary to avoid no contact areas with the implant. After appropriate femoral sizing, different from the original technique, we start from chamfer resections and AP are made later on. We believe the “chamfer first” technique is crucial to avoid imperfect femur resection, which can be the reason why the implant does not seat very well into the bone.

AP resections are parallel cuts and they are less sensitive to small micro-movements of the jig. After that, we complete the posterior condylar resection, changing the saw with a thinner one for the posterolateral bone resection in order to save the popliteus ligament.

The next step is the research of the optimal fit of the baseplate in tibial sizing, close to the cortical ream. After tibial sizing, we complete the tibia by reverse drilling where the bone is softer and normal drilling where the bone is harder. At the end, the impaction of the tibia baseplate should be symmetric: medial and lateral, anterior, and posterior. During the femur implantation, the surgeon must raise the hand while pushing the femoral component. The aim is to achieve no space area around the corner of the femoral component prosthesis, even if less than 2 mm of gap is accepted. Knee stability is tested throughout a complete ROM. At last, we use the intraoperative “pull-out lift-off” (POLO) test to check the appropriate tension of PCL.

### 1.8. Short Term Follow Up of a Novel 3D Printed Cementless TKA

**Figure 2:**
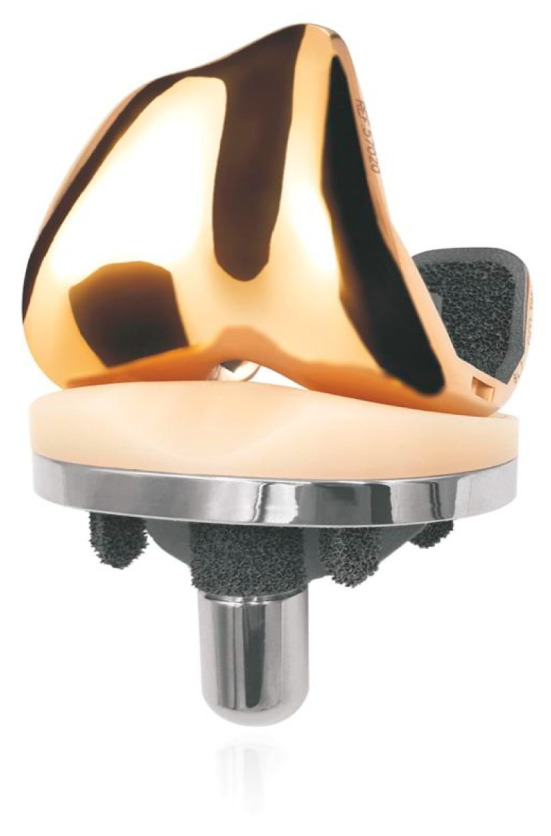
GKS Prime Flex Traser (Permedica, Merate, Italy).

Among the 370 primary total knee replacements performed in the period 2021 to 2022 at our institution, 127 received GKS prosthesis and they were included in a perspective study, 60 patients received a 3D-printed cementless TKA (Figure 2). All the patients were evaluated at 3, 6, and 12 months by recording the VAS score, the Oxford Knee Score, the Knee Society Score, and the Forgotten Knee Score. No significant differences between the two groups were reported. The mean time to reach a VAS score < 3 was 6 months in 70% of the patients. The mean FJS was 67 at 3 months, 76 at 6 months, and 79 at 12 months post-operatively.

### 1.9. Cost Analysis

Cementless implants are surely more expensive than their cemented counterparts, potentially creating an obstacle to their diffusion in a cost-sensitive health system. Moreover, considering that prosthetic implants account for the single largest expense in the 90-day episode of care for TKA, making up about 25% of the total cost, the use of higher-cost implants may be limited or restricted [61]. However, Laurie et al. [62] compared 80 cementless and 67 cemented single-design TKA and showed that although the general cost of cemented TKA implants is lower than the cementless, the actual cost of the procedure is less for the press-fit technique when considering the costs of operating theatre time, cement, and cementing accessories. Indeed, despite the increased charge of USD 366 between the two implants, the authors reported longer operative time for cemented TKA (11.6 min at USD 35 per minute; *p* = 0.001) with cement and accessories costs ranging from USD 170 to USD 625 reaching an additional cost related to the cementation of USD 588 to USD 1043. Similar findings were reported by Yayac et al. [63] among 2426 TKA, with higher cementless implant costs (USD 3047.80 vs. USD 2808.73, *p* < 0.0001) but lower supply costs (USD 639.49 vs. USD 815.57, *p* < 0.0001) and lower operating room personnel costs (USD 982.01 vs. USD 1238.26, *p* < 0.0001) outlined that, at their institution, cementless TKA did not significantly increase total procedural costs when compared to traditional cemented TKA. Conversely, Gwam et al. [64] reported on a National Inpatient Sample (NIS) analysis of 167,930 TKAs that cementless TKA (4870) was associated with higher inpatient hospital costs (USD 17,357 vs. USD 16,888) and charges (USD 67,366 vs. USD 64,190; *p* < 0.001), despite its association with a lower mean length of stay (2.63 vs. 2.71 days; *p* < 0.001), and higher odds of being discharged to home (OR = 1.99; *p* = 0.002).

## 2. Conclusions

There are large amounts of proof that innovations in technology and design have helped modern cementless TKA implants improve primary stability in both the femur and tibia. This means that short-term and mid-term revision rates are comparable to cemented implants [65]. All of these positive prognostic factors may lead orthopedic surgeons into a future where cementless fixation may be considered the gold-standard technique in TKA in young and active patients.

## Data Availability

Data supporting this study are openly available on PubMed.
